# Acute Effects of Summer Air Pollution on Pulmonary Function and Airway Inflammation in Healthy Young Women

**DOI:** 10.2188/jea.JE20130155

**Published:** 2014-07-05

**Authors:** Yoshiko Yoda, Naruhito Otani, Shiro Sakurai, Masayuki Shima

**Affiliations:** 1 Department of Public Health, Hyogo College of Medicine, Nishinomiya, Hyogo, Japan; 1 兵庫医科大学公衆衛生学; 2 Department of Environmental Science, School of Social Information Studies, Otsuma Women’s University, Tama, Tokyo, Japan; 2 大妻女子大学社会情報学部環境情報学専攻

**Keywords:** airway inflammation, exhaled breath condensate, ozone, particulate matter, pulmonary function

## Abstract

**Background:**

Exposure to air pollution has been reported to be associated with asthma exacerbation. However, little is known about the effects of air pollutant exposure in healthy people. A panel study was conducted to evaluate the acute effects of air pollutants on pulmonary function and airway inflammation in healthy subjects.

**Methods:**

Exhaled breath condensate (EBC) pH, fractional concentration of exhaled nitric oxide (FeNO), and pulmonary function were measured in 21 healthy young women repeatedly for two weeks in the summer in Tokyo, Japan. The concentrations of air pollutants were obtained from the monitoring stations in the neighborhoods where the subjects lived. Statistical analyses were performed using generalized estimating equations.

**Results:**

EBC pH decreased significantly with a 10-ppb increase in the 4-day average ozone (O_3_) concentration and a 10-µg/m^3^ increase in the 4-day average suspended particulate matter (SPM) concentration (−0.07 [95% confidence interval {CI} −0.11 to −0.03] and −0.08 [95% CI −0.12 to −0.03], respectively). Subjects with a history of rhinitis showed marked decreases in EBC pH associated with increases in O_3_ and SPM. The changes in forced expiratory volume in 1 second (FEV_1_) were also significantly associated with a 10-µg/m^3^ increase in the 3-day average concentration of SPM (−0.09 L [95% CI −0.17 to −0.01]). FeNO increased significantly in relation to the increase in O_3_ and SPM among only subjects with a history of asthma.

**Conclusions:**

Over the course of the study, EBC became significantly acidic with increases in O_3_ and SPM concentrations. Furthermore, higher SPM concentrations were associated with decreased FEV_1_. Subjects with a history of rhinitis or asthma are considered to be more susceptible to air pollutants.

## Abstract (ja)

**背景：:**

大気汚染への曝露が喘息患者の症状悪化と関連していることは数多く報告されている。しかし、健常者に対する大気汚染の急性影響はほとんど知られていない。そこで、健常成人を対象にパネル研究を実施し、肺機能と気道炎症に対する大気汚染物質の急性影響を評価した。

**方法：:**

東京都内の健常な女子学生21名を対象として、呼気凝縮液（EBC）のpH、呼気一酸化窒素濃度（FeNO）及び肺機能の測定を夏季の2週間の間に繰り返し実施した。大気汚染物質の濃度は、対象者が居住する近隣の測定局における浮遊粒子状物質（SPM）及びオゾン（O_3_）濃度を用いて、各指標との関連を一般化推定方程式により解析した。

**結果：:**

EBC pHは、検査前4日平均のO_3_濃度10ppb増加あたり-0.07 [95％信頼区間：-0.11,-0.03]、検査前4日平均のSPM濃度10 μg/m^3^増加あたり-0.08 [95％信頼区間：-0.12,-0.03]であり、いずれも有意な低下が観察された。鼻炎の既往があるものは、O_3_及びSPM濃度の増加によりEBC pHの顕著な低下がみられた。1秒量（FEV_1_）も検査前3日平均のSPM濃度と有意に関連しており、10 μg/m^3^増加あたり-0.09 [95%信頼区間：-0.17, -0.01] Lの低下であった。FeNOは、喘息の既往のあるものにおいてのみO_3_及びSPM濃度が増加すると有意に増加した。

**結論：:**

研究期間中に大気中のO_3_及びSPM濃度が上昇すると、EBC pHが有意に酸性になることが明らかとなった。また、SPMが高濃度ではFEV_1_の低下との関連があった。鼻炎または喘息の既往があるものは大気汚染物質の影響を受けやすいと考えられた。

## INTRODUCTION

The acute effects of air pollution on respiratory symptoms have been widely reported.^1^^,^^2^ Many studies have shown that ambient air pollution, especially particulate matter, is associated with effects on respiratory symptoms and exacerbation of asthma.^3^^–^^9^ Ozone (O_3_) is also a major air pollutant, and studies have reported that high concentrations of ozone in summer affect respiratory symptoms in subjects with asthma.^10^^–^^13^ Berhane et al showed that a short-term increase in O_3_ concentration was related to airway inflammation in children.^14^ In addition, elevated O_3_ levels were reported to be associated with airway inflammation in atopic participants, both with and without asthma.^15^ While some findings have shown the acute effects of O_3_ on respiratory symptoms, the pathophysiological mechanisms for the effects of O_3_ are not fully understood.

In recent years, the measurement of the fractional concentration of nitric oxide in exhaled air (FeNO) has become valuable for the noninvasive and quantitative assessment of airway inflammation.^16^ Exhaled breath condensate (EBC) is also used as an indicator of oxidative stress.^17^ Since these assays are simple and can be performed repeatedly, they are considered to be useful for evaluating airway inflammation in epidemiologic studies. Using these methods, the acute effects of air pollutants on airway inflammation have been evaluated in asthmatic children and adolescents.^18^^,^^19^

Most previous studies have reported the acute effects of air pollution in children and patients with asthma, but few studies have investigated the effects in healthy adults. The aim of this study was to investigate whether rising concentrations of air pollutants have an acute effect on the respiratory system in healthy adult subjects. Therefore, tests of pulmonary function and the inflammatory condition of the airways were repeatedly performed in 21 healthy female students, and the potential effects of air pollution in the summer season, when a large variation in the concentrations of air pollutants was forecasted, were evaluated.

## METHODS

### Study design and subjects

A panel study was conducted for two weeks in July 2012. The subjects of this study were 21 healthy female students, aged 20–23 years, who had to commute to a university in Tokyo, Japan. They were recruited from among non-smoking students who attended several seminars in their third or fourth year. The university is located in a suburban area in Tokyo, and all of the study subjects lived in the Tokyo metropolitan area. The study protocol was approved by the Ethics Committee of Hyogo College of Medicine. The objective and method of this study were fully explained to the subjects, and written informed consent was obtained from each subject before the study.

At the beginning of the study, respiratory symptoms, medical history, family history, and smoking habits were evaluated by a modified ATS-DLD-78 questionnaire.^20^ The subjects were also asked to record their daily movements during the study period. Measurements of pulmonary function and airway inflammation were repeated four times during the study period and were performed on the same day for all subjects. Subjects were requested to refrain from eating and drinking for 2 hours before measurements in order to not affect the results of the tests. The order of testing was FeNO measurement, pulmonary function tests, and collection of EBC.

### Sampling of exhaled air and measurement of FeNO

The FeNO levels were measured using the off-line method according to ATS/ERS recommendations.^16^ The subjects inspired maximally in the seated position, and then they exhaled into a 1.5-L Mylar bag (Sievers Instruments, Inc., Boulder, CO, USA) to keep the mouth pressure constant at 15 cmH_2_O and the expiratory flow rate at 50 mL/s. Exhaled air of the first 10 seconds (500 mL) was discarded to prevent contamination by NO from the nasal cavity and upper airway. The collection was repeated three times for each subject. The bag samples were stored at 4°C until analysis, and FeNO was measured using a chemiluminescence NO analyzer (Model NA-623N; Kimoto Electric Co., Ltd., Osaka, Japan) within 24 hours after collection.^21^ The analyzer was calibrated daily using zero-gas and standard NO gas (735 ppb). The values of FeNO are shown as the means of three samples. If the deviation for each set of triplicate samples was larger than 20% of the mean, the highest FeNO value was removed from the calculation of the mean.

### Pulmonary function tests

Pulmonary function tests were performed using an electronic expiratory flow meter (Vitalograph 2110; Vitalograph Ltd., Buckingham, UK). Before beginning the testing, the device was calibrated using a 3-L syringe. Peak expiratory flow (PEF) and forced expiratory volume in 1 second (FEV_1_) were measured four times for all subjects. For each measurement, the highest PEF and FEV_1_ values were selected from more than two reproducible maneuvers.

### Exhaled breath condensate collection

EBC was collected using an RTube device (Respiratory Research Inc., Charlottesville, VA, USA) during 15 min of quiet breathing while wearing a nose clip. Approximately 1.5 mL of EBC sample was obtained. Immediately after collection, the obtained sample was divided into two aliquots. One sample was degassed with argon gas at a flow rate of 300 mL/min bubbling. The pH of EBC was then measured using a pH meter (F-52; HORIBA, Ltd., Kyoto, Japan). Another sample was stored at −80°C until measurement of 8-isoprostane, which is an oxidative stress marker. 8-isoprostane concentrations were measured using a specific enzyme-linked immunosorbent assay (Cayman Chemical Company, Ann Arbor, MI, USA). The lower limit of detection of this assay was 2.7 pg/mL. Unfortunately, the values of 8-isoprostane in EBC were below the detection limit in most samples, although the values were detected in some samples. Thus, the 8-isoprostane values could not be used as an oxidative stress marker.

### Air pollution monitoring

The concentrations of air pollutants and the meteorological data at the monitoring stations in the neighborhoods where the subjects lived were obtained from the Atmospheric Environmental Regional Observation System of the Ministry of the Environment, Japan. If the meteorological data were unavailable at the monitoring stations, they were obtained from the Japan Meteorological Agency. The 24-hour averages of suspended particulate matter (SPM), nitrogen dioxide (NO_2_), and O_3_ concentrations, temperature, and relative humidity were used. In Japan, SPM is defined under the Japanese Air Quality Standard as particles with an aerodynamic diameter of ≤10 µm by the 100% cutoff point, which corresponds approximately to particulate matter <7 µm in aerodynamic diameter by the 50% cutoff point.^22^ The distances between the subjects’ residences and the monitoring stations were 0.2 to 8.3 km, and there was no significant difference in the surrounding environment.

### Statistical analysis

Since the FeNO level was roughly log-normally distributed, logarithms of the measurements were used for analysis. The measurements of EBC pH, log FeNO, PEF, and FEV_1_ were examined in relation to the concentrations of air pollutants. For regression analyses of the measurements, the Generalized Estimating Equation (GEE)^23^ was used, which is suitable for correlated data in individual repeated measures. The standard error of the regression estimate was adjusted for possible correlation among outcomes from a single subject. After adjusting for temperature and relative humidity, we calculated mean changes and 95% confidence intervals (CIs) in EBC pH, FeNO, PEF, and FEV_1_ for 10-µg/m^3^ increases in SPM or 10-ppb increases in O_3_ or NO_2_.

The average concentrations of each pollutant during the 24 hours preceding measurement were used as exposure variables. In order to assess the potentially delayed effects of air pollutants, the concentration for the previous day and 2- to 5-day averages were also examined. Next, because the sensitivity to air pollutants may differ in relation to respiratory symptoms, the participants were stratified by the presence or absence of a history of rhinitis or asthma. The changes in EBC pH, FeNO, PEF, and FEV_1_ associated with 4-day averages of O_3_ and SPM were estimated, because they showed the greatest changes in the analyses involving all subjects. Furthermore, the analyses were performed using two-pollutant models to adjust for potential confounding effects of co-pollutants using the 4-day averages of each pollutant.

All statistical analyses were performed using SPSS version 19 (IBM Co., Armonk, NY, USA). A two-tailed value of *P* < 0.05 was considered significant.

## RESULTS

**Figure 1.**
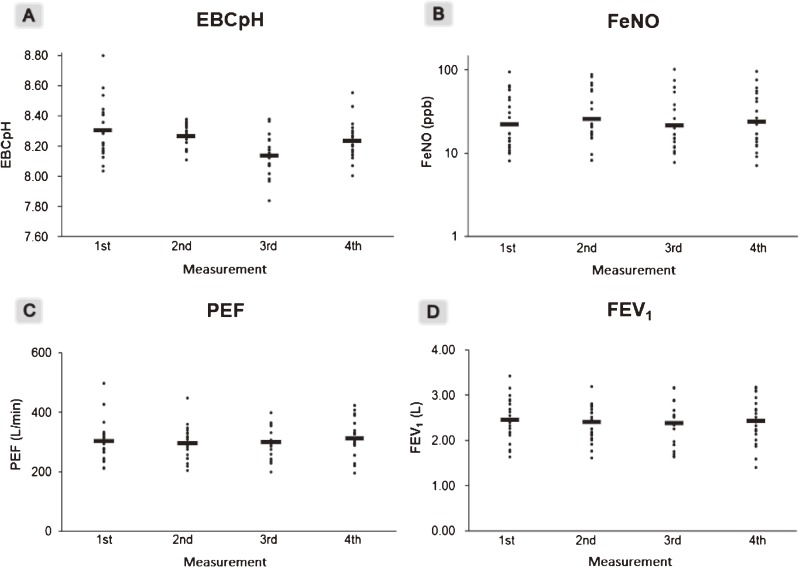
Distributions of the results of EBC pH (A), FeNO (B), PEF (C), and FEV_1_ (D) for each of the four measurements. Horizontal bars represent the means of each test. The results of FeNO (B) are plotted on a logarithmic scale, and the bars show the geometric means.

Table 1 presents the characteristics of the study subjects. A total of 21 healthy non-smoking subjects took part in the study. The results of pulmonary function tests and airway inflammation tests are shown in Figure 1. A total of 84 samples of FeNO, PEF, and FEV_1_ were collected, and 83 samples of EBC were collected, because one sample was contaminated by saliva. The average EBC pH was slightly lower in the samples collected the third time than at other times. The average PEF was slightly lower the second time and highest the fourth time.

**Table 1.** Characteristics of study subjects

	Total (*n* = 21)
Age, mean (SD) (years)	21.0 (0.6)
Height, mean (SD) (cm)	158.8 (5.9)
History of asthma (yes/no)	4/17
History of rhinitis (yes/no)	12/9
History of pollinosis (yes/no)	8/13

SD, Standard deviation.

**Figure 2.**
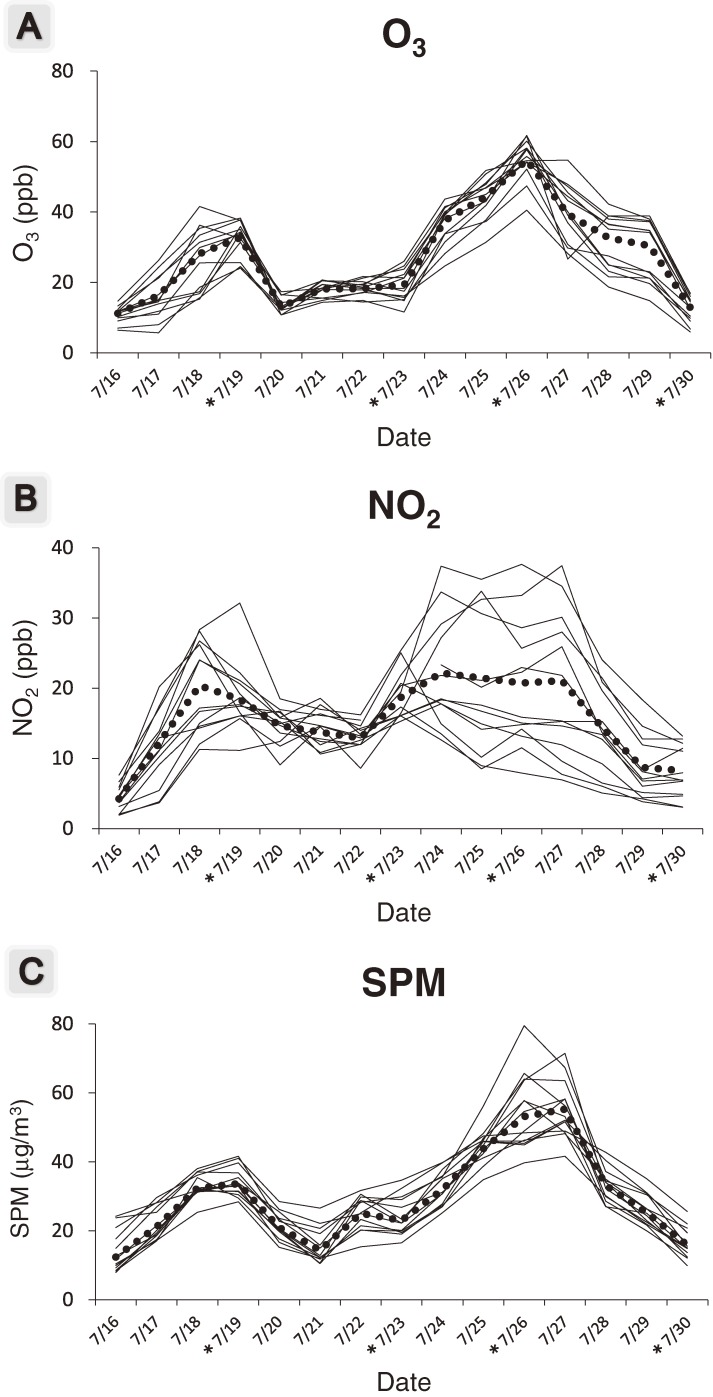
The 24-hour mean concentrations of O_3_ (A), NO_2_ (B), and SPM (C) for each subject during the study period. The dotted lines show the daily average concentrations of each pollutant for all subjects. *Respiratory function tests were conducted on these days.

Daily average concentrations of air pollutants during this study period are shown in Figure 2. The concentrations of O_3_ and SPM were highest on 26 July, when the third pulmonary function test was conducted. The concentration of NO_2_ differed considerably by the residence of the subjects, but the concentrations of O_3_ and SPM did not differ by the subjects’ residence. Table 2 shows the correlation coefficients of the concentrations of air pollutants; concentrations of O_3_, NO_2_, and SPM were correlated with each other.

**Table 2.** Correlation coefficients of air pollutants and meteorological factors

	O_3_	NO_2_	SPM	Temperature	Relative humidity
O_3_	1	0.715*	0.909*	0.459	−0.286
NO_2_		1	0.752*	0.042	0.076
SPM			1	0.431	−0.227
Temperature				1	−0.925*
Relative humidity					1

O_3_, ozone; NO_2_, nitrogen dioxide; SPM, suspended particulate matter.

Pearson’s correlation coefficients were calculated using the means of daily average concentrations of air pollutants or meteorological factors for each subject.

*P < 0.01.

Table 3 shows the changes in EBC pH, FeNO, PEF, and FEV_1_ associated with a 10-µg/m^3^ or 10-ppb increase in the concentration of each pollutant, using single-pollutant models adjusted for temperature and relative humidity. The change in EBC pH was significantly negatively associated with the concentrations of both O_3_ and SPM; in particular, the change was greatest in relation to the 4-day average concentration of O_3_ and SPM (−0.07 [95% CI −0.11 to −0.03] and −0.08 [95% CI −0.12 to −0.03], respectively). The change in FeNO was not associated with O_3_, NO_2_, or SPM. The change in FEV_1_ was negatively associated with the 3-day average concentration of SPM (−0.09 L [95% CI, −0.17 to −0.01]). PEF was not significantly associated with any pollutants. The results of the analyses excluding the four subjects who lived more than 5 km from the monitoring stations are shown in eTable 1. The effects of O_3_ on EBC pH were similar to the results of the analyses of all subjects. In addition, as a sensitivity analysis, the data were reanalyzed excluding outliers for each pulmonary function. The results showed similar patterns of the effects of pollutants as with the full data set (results not shown).

**Table 3.** Estimated changes and 95% CIs in respiratory function test results per 10-µg/m^3^ or 10-ppb increase in each pollutant during the study period

	EBC pH			log FeNO			PEF (L/min)			FEV_1_ (L)		
			
	Change	95% CI	*P* value	Change	95% CI	*P* value	Change	95% CI	*P* value	Change	95% CI	*P* value
O_3_												
Test day^a^	−0.02	(−0.04, 0.00)	0.024	0.02	(−0.05, 0.08)	0.623	−3.16	(−9.71, 3.40)	0.346	−0.02	(−0.06, 0.03)	0.489
Previous day^b^	−0.04	(−0.08, 0.00)	0.026	0.07	(−0.11, 0.25)	0.427	2.82	(−14.64, 20.29)	0.752	0.00	(−0.15, 0.15)	0.977
2-day average^c^	−0.04	(−0.06, −0.01)	0.014	0.03	(−0.08, 0.13)	0.612	−1.61	(−12.24, 9.01)	0.766	−0.01	(−0.09, 0.07)	0.783
3-day average^d^	−0.05	(−0.09, −0.02)	0.003	0.02	(−0.10, 0.15)	0.711	1.31	(−10.57, 13.19)	0.829	0.00	(−0.10, 0.10)	0.948
4-day average^d^	−0.07	(−0.11, −0.03)	0.001	0.00	(−0.18, 0.18)	0.999	5.64	(−8.69, 19.98)	0.440	0.01	(−0.12, 0.14)	0.880
5-day average^d^	−0.06	(−0.11, −0.02)	0.003	−0.04	(−0.23, 0.15)	0.659	12.14	(−3.45, 27.74)	0.127	0.04	(−0.10, 0.18)	0.610
NO_2_												
Test day^a^	−0.01	(−0.04, 0.01)	0.375	−0.13	(−0.34, 0.08)	0.214	−6.23	(−22.17, 9.71)	0.444	0.04	(−0.12, 0.21)	0.618
Previous day^b^	0.01	(−0.02, 0.03)	0.614	−0.07	(−0.26, 0.13)	0.517	−13.13	(−28.99, 2.72)	0.104	0.03	(−0.12, 0.18)	0.671
2-day average^c^	0.00	(−0.03, 0.02)	0.893	−0.10	(−0.31, 0.12)	0.373	−10.77	(−27.60, 6.06)	0.210	0.04	(−0.13, 0.20)	0.645
3-day average^d^	−0.01	(−0.04, 0.02)	0.492	−0.11	(−0.37, 0.16)	0.430	−13.55	(−33.87, 6.78)	0.192	0.05	(−0.15, 0.24)	0.656
4-day average^d^	−0.01	(−0.05, 0.03)	0.479	−0.21	(−0.52, 0.11)	0.200	−5.41	(−30.41, 19.59)	0.672	0.10	(−0.13, 0.33)	0.401
5-day average^d^	−0.01	(−0.06, 0.04)	0.684	−0.31	(−0.67, 0.04)	0.084	−0.14	(−30.78, 30.49)	0.993	0.14	(−0.13, 0.40)	0.314
SPM												
Test day^a^	−0.02	(−0.04, 0.00)	0.019	−0.01	(−0.06, 0.05)	0.848	−2.53	(−10.29, 5.22)	0.522	−0.04	(−0.08, 0.01)	0.129
Previous day^b^	−0.05	(−0.08, −0.02)	0.001	0.03	(−0.08, 0.15)	0.573	−5.87	(−18.45, 6.71)	0.360	−0.07	(−0.15, 0.01)	0.096
2-day average^c^	−0.03	(−0.05, −0.01)	0.002	0.00	(−0.07, 0.07)	0.999	−3.64	(−12.89, 5.62)	0.441	−0.05	(−0.11, 0.00)	0.057
3-day average^d^	−0.06	(−0.09, −0.02)	0.001	0.02	(−0.09, 0.12)	0.776	−3.83	(−17.74, 10.09)	0.590	−0.09	(−0.17, −0.01)	0.034
4-day average^d^	−0.08	(−0.12, −0.03)	0.002	−0.04	(−0.19, 0.12)	0.654	2.82	(−20.26, 25.90)	0.811	−0.13	(−0.29, 0.02)	0.093
5-day average^d^	−0.07	(−0.11, −0.02)	0.006	−0.08	(−0.25, 0.09)	0.337	9.62	(−15.47, 34.70)	0.452	−0.10	(−0.29, 0.08)	0.267

CI, confidence interval; O_3_, ozone; NO_2_, nitrogen dioxide; SPM, suspended particulate matter.

^a^Test day: The 24-hour mean concentration of a pollutant collected on the day that respiratory function tests were done.

^b^Previous day: The 24-hour mean concentration of a pollutant collected on the day before the respiratory function tests were done.

^c^2-day average: The mean concentration of a pollutant collected on the day and the day before the respiratory function tests were done.

^d^3- to 5-day average: The mean concentration of a pollutant collected on the day and the 2 to 4 days before the respiratory function tests were done.

All models are adjusted for temperature and relative humidity.

**Figure 3.**
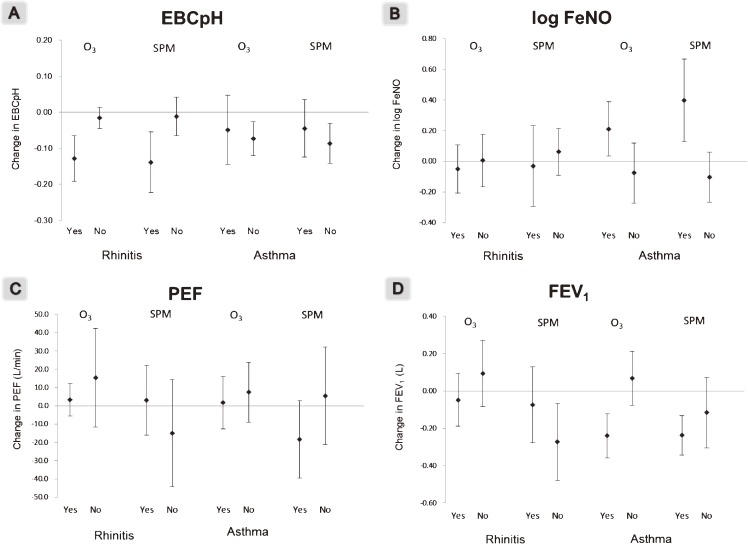
Estimated changes and 95% CIs in EBC pH (A), log FeNO (B), PEF (C), and FEV_1_ (D) with increases in air pollutants. The concentrations of the pollutants were averaged over 4 days, including the day of and 3 days before the respiratory function tests.

After the participants were stratified by the presence or absence of a history of rhinitis or asthma, the changes in EBC pH, FeNO, PEF, and FEV_1_ in relation to increases in the 4-day average concentration of air pollutants are shown in Figure 3. Among subjects who had a history of rhinitis, increases in O_3_ and SPM concentrations were significantly associated with low EBC pH. However, no association was seen in subjects without a history of rhinitis. On the other hand, FEV_1_ decreased significantly with increases in SPM concentrations among subjects without a history of rhinitis. EBC pH significantly decreased with increases in O_3_ or SPM concentrations only among subjects without a history of asthma (Figure 3a). FeNO levels were significantly higher with increases in O_3_ and SPM concentrations among subjects with a history of asthma (Figure 3b). PEF was associated with neither O_3_ nor SPM, regardless of the presence of rhinitis or asthma (Figure 3c). FEV_1_ showed significant decreases with high concentrations of O_3_ and SPM only among subjects with a history of asthma (Figure 3d).

Two-pollutant models were also tested to adjust for confounding effects of co-pollutants. Table 4 shows the changes in EBC pH, FeNO, PEF, and FEV_1_ associated with the 4-day average concentrations of each pollutant. EBC pH showed a significant decrease in relation to 4-day average concentrations of O_3_ after adjustment for NO_2_ or SPM concentrations, as well as in the single-pollutant models. On the other hand, the association between EBC pH and SPM concentration was not significant after adjustment for O_3_ concentrations, while the associations were significant in the single- and two-pollutant model with SPM and NO_2_. The associations of the other outcomes with the 4-day average concentrations of air pollutants were not significant in either the single- or two-pollutant models.

**Table 4.** Estimated changes and 95% CIs in respiratory function test results per 10-µg/m^3^ or 10-ppb increase in each single- and two-pollutant model

	EBC pH			log FeNO			PEF (L/min)			FEV_1_ (L)		
			
	Change	95% CI	*P* value	Change	95% CI	*P* value	Change	95% CI	*P* value	Change	95% CI	*P* value
O_3_												
Single	−0.07	(−0.11, −0.03)	0.001	0.00	(−0.18, 0.18)	0.999	5.64	(−8.69, 19.98)	0.440	0.01	(−0.12, 0.14)	0.880
+NO_2_	−0.07	(−0.11, −0.03)	0.001	0.02	(−0.19, 0.23)	0.851	6.27	(−9.82, 22.35)	0.445	0.00	(−0.15, 0.15)	0.991
+SPM	−0.05	(−0.09, −0.01)	0.008	0.03	(−0.23, 0.29)	0.837	6.73	(−21.78, 35.24)	0.644	0.12	(−0.09, 0.32)	0.277
NO_2_												
Single	−0.01	(−0.05, 0.03)	0.479	−0.21	(−0.52, 0.11)	0.200	−5.41	(−30.41, 19.59)	0.672	0.10	(−0.13, 0.33)	0.401
+O_3_	0.00	(−0.05, 0.05)	0.979	−0.21	(−0.56, 0.14)	0.242	−6.66	(−33.73, 20.42)	0.630	0.10	(−0.16, 0.36)	0.448
+SPM	0.01	(−0.02, 0.04)	0.456	−0.21	(−0.59, 0.16)	0.266	−6.97	(−34.69, 20.76)	0.622	0.16	(−0.10, 0.42)	0.236
SPM												
Single	−0.08	(−0.12, −0.03)	0.002	−0.04	(−0.19, 0.12)	0.654	2.82	(−20.26, 25.90)	0.811	−0.13	(−0.29, 0.02)	0.093
+O_3_	−0.04	(−0.08, 0.00)	0.078	−0.06	(−0.31, 0.20)	0.666	−2.27	(−41.99, 37.45)	0.911	−0.22	(−0.47, 0.03)	0.088
+NO_2_	−0.08	(−0.13, −0.03)	0.001	0.02	(−0.20, 0.24)	0.876	4.58	(−19.86, 29.02)	0.713	−0.17	(−0.37, 0.02)	0.079

CI, confidence interval; O_3_, ozone; NO_2_, nitrogen dioxide; SPM, suspended particulate matter.

The concentrations of pollutants were averaged over 4 days including the same day and the 3 days before the respiratory function tests were done.

All models are adjusted for temperature and relative humidity.

## DISCUSSION

In this panel study, increases in O_3_ and SPM in the neighborhood of the subjects’ residence were significantly associated with airway inflammation, as reflected by decreases in EBC pH. Furthermore, increase in the 3-day average concentrations of SPM was associated with a decrease in FEV_1_. Inhalation of O_3_ causes airway inflammation, wheezing, dyspnea, and damage to pulmonary function.^24^ It has been reported that an increase in O_3_ concentrations was associated with a decrease in EBC pH,^19^ which is consistent with the present results. Furthermore, exposure to particulate matter is known to affect pulmonary dysfunction.^25^ However, conflicting findings have also been reported. For example, Maestrellie et al reported that no associations were found between short-term exposure to particulate matter and lung function or inflammatory responses in asthmatic adults.^26^ In the present study, increased SPM was significantly associated with decreased EBC pH and FEV_1_ in healthy subjects. For NO_2_, there was no significant association with any measure in the present study. The previous studies reported that EBC pH among adolescents and asthmatic adults was not affected even by NO_2_ concentrations higher than those observed in this study.^19^^,^^26^

Findings on the short-term effects of air pollutants on airway inflammation markers have not always been consistent. The present study results showed that the changes in EBC pH were greater in relation to the 4-day average concentration of O_3_ and SPM than to the concentrations on the same day or the previous day, suggesting the strong effects of the 4-day average concentration of these air pollutants on the respiratory system. Patel et al reported that multiday average concentrations of black carbon were more strongly associated with EBC pH than shorter averaging times.^19^ Exposures accumulated over multiple days may have a greater effect on airway inflammation. The temporal relationship between exposure to air pollution and airway inflammation should be further evaluated.

In the present study, the daily average concentrations of O_3_ and SPM changed markedly during the study period, but the time courses of each pollutant were similar across the participants. Therefore, these pollutants were considered to be spread widely over the Tokyo metropolitan area. On the other hand, the concentrations of NO_2_ differed among participants, which is suggestive of the effects of local emissions.

FEV_1_ has been widely used as a method for evaluating the effect of air pollutants on lung function. Previous studies reported that decreases in FEV_1_ were associated with increased exposure to particulate matter.^27^^,^^28^ In the present study, FEV_1_ was negatively associated with SPM concentrations, and the association was strongest among subjects with a history of asthma. This result is considered to be consistent with the findings of Delfino et al,^3^ who reported stronger associations between particulate matter exposures and FEV_1_ decrements among children with asthma.

EBC can be noninvasively collected to evaluate airway inflammation,^17^ and EBC pH has been proposed as a biomarker of inflammation that reflects the acid-base balance of the airway, which is regulated primarily by epithelial ammonia production and lining fluid proton-buffering.^29^ Vaughan et al demonstrated that measurement of EBC pH is a simple, robust, and reproducible assay and a relevant marker of airway inflammation.^30^ EBC pH values have been reported to be significantly lower in patients with moderate asthma than in mild asthma patients and control subjects.^31^ Recently, Patel et al found that increases in black carbon concentrations were associated with decreases in EBC pH.^19^

In the present study, EBC pH was significantly lower with increased O_3_ or SPM concentrations, and the associations were prominent among subjects with a history of rhinitis. Subjects with a history of rhinitis may also have airway inflammation along with nasal mucosal inflammation. On the other hand, decreases in EBC pH were significantly associated with increases in O_3_ and SPM concentrations, independent of a history of asthma. Epton et al reported that there was no effect of air pollution on EBC pH in patients with asthma and healthy subjects, although they measured EBC pH immediately after collection without degassing.^4^ In the present study, EBC pH was measured after degassing with argon, and the effects by air pollutants were observed even among healthy subjects without asthma.

8-Isoprostane has been used as a marker of oxidative stress.^32^ In the present study, however, values of 8-isoprostane in EBC were below the detection limit in most samples. Because the subjects of our study were healthy young women with little sign of airway inflammation, it may not have been possible to detect 8-isoprostane in their EBC.

FeNO is produced by inducible nitric oxide synthase in the bronchial epithelia constituting the mucous membranes of the airway.^33^ The measurement of FeNO concentrations is used as a method of assessing airway inflammation.^34^^,^^35^ In previous studies, it has been reported that increases in FeNO concentrations are associated with exposure to air pollution.^8^ In the present study, FeNO concentrations increased significantly with increased O_3_ and SPM concentrations only among subjects with a history of asthma, despite the limited number of subjects with such a history. This result was similar to the results of a previous study.^15^ On the other hand, Modig et al recently reported that the effect of NO_2_ on FeNO was stronger than the effect of O_3_ in subjects with asthma.^36^

In contrast, we found that FeNO concentrations decreased with increases in NO_2_ concentrations. This result is inconsistent with those of Delfino et al, who reported that FeNO concentrations increased with increases in NO_2_ concentration.^37^ This discrepancy may be due to differences in the variation of NO_2_ concentrations during the study period. In the present study, NO_2_ concentrations varied among study areas, but the change in the concentrations during the study period was relatively small for most subjects. It is possible that the effects of air pollutants on FeNO differ in relation to the type of air pollutants and exposure conditions.

The present study has several limitations. First, the concentrations of air pollutants in the monitoring stations in the vicinity of the subjects’ residences were used to assess exposure to air pollutants. Therefore, it was not possible to accurately assess the subjects’ personal exposure levels. Delfino et al suggested that ambient levels of air pollutants might not accurately reflect the exposure to pollutants caused by fossil fuel combustion.^37^ However, the subjects in this study attended the same university, and a large difference was not observed in the variation of concentrations of air pollutants in the subjects’ residential areas. Furthermore, all of the subjects were also confirmed through their daily records to have stayed in the Tokyo metropolitan area throughout the study period. Second, the study period was only two weeks, and the changes in pulmonary function and airway inflammation may have been physiological. However, O_3_ and SPM concentrations changed markedly during the period, and EBC pH decreased in relation to the increase in O_3_ and SPM concentrations. Third, the statistical power was not adequate due to the limited number of subjects. However, all 21 subjects participated in four measurements during the study period, and the results of 84 measurements were evaluated. Therefore, the acute effects of O_3_ and SPM were observed despite the relatively small number of subjects. Fourth, it was not possible to evaluate the interaction by respiratory symptoms of the subjects. Respiratory symptoms, such as cough, phlegm, and wheezing, were evaluated by a standard questionnaire, but few subjects had such symptoms because they were healthy students.

In the present study, healthy young adult women were the target subjects. We believe that all people in urban districts may suffer the effects of air pollution on health in daily life. Therefore, we would like to perform a study in a larger, more varied population of healthy subjects. In particular, the effects of air pollutants among males should be evaluated, because the subjects of this study were only females.

In conclusion, the present study showed that increases in O_3_ or SPM concentrations were associated with decreased pulmonary function and decreased EBC pH. Subjects with a history of rhinitis showed greater changes in EBC pH with increases in O_3_ and SPM concentrations. In addition, subjects with a history of asthma showed higher FeNO levels with increased O_3_ concentrations. These results suggest that subjects with a history of rhinitis or asthma are especially susceptible to the harmful effects of air pollution. Because this study involved a small number of subjects, the effects of short-term exposure to air pollutants should be further evaluated in a larger study.

## ONLINE ONLY MATERIALS

eTable 1. Estimated changes and 95% CIs in the respiratory function test results per 10-µg/m^3^ or 10-ppb increase in each pollutant during the study period among the 17 subjects who lived less than 5 km from the monitoring stations.

Abstract in Japanese.
