# Supplementary material for: Acute Effects of Summer Air Pollution on Pulmonary Function and Airway Inflammation in Healthy Young Women
Source: J Epidemiol. 2014 Jul 5;24(4):312–20. doi: 10.2188/jea.JE20130155 (PMC4074636; doi:10.2188/jea.JE20130155)
Supplement: eTable 1. [file je-24-312-s001.pdf]

e**Table 1.** Estimated changes and 95% CIs in the respiratory function test results per 10-μg/m^3 or 10-ppb increase in each pollutant during the study period among the 17 subjects who lived less than 5 km from monitoring stations

	EBC pH			log FeNO			PEF (L/min)			FEV_1 (L)		
	Change	95% CI	*P* Value	Change	95% CI	*P* Value	Change	95% CI	*P* Value	Change	95% CI	*P* Value
O_3												
Test day[a]	-0.02	(-0.04, 0.00)	0.042	0.01	(-0.07, 0.08)	0.836	-4.64	(-10.33, 1.06)	0.111	-0.03	(-0.08, 0.02)	0.197
Previous day[b]	-0.03	(-0.06, 0.01)	0.118	0.09	(-0.12, 0.30)	0.416	-4.87	(-17.64, 7.90)	0.455	-0.04	(-0.21, 0.14)	0.678
2-day average[c]	-0.03	(-0.06, 0.00)	0.044	0.02	(-0.10, 0.14)	0.688	-5.58	(-13.36, 2.21)	0.160	-0.04	(-0.12, 0.05)	0.383
3-day average[d]	-0.04	(-0.08, -0.01)	0.026	0.02	(-0.13, 0.17)	0.759	-3.63	(-11.40, 4.14)	0.360	-0.03	(-0.14, 0.07)	0.540
4-day average[d]	-0.05	(-0.10, -0.01)	0.027	0.00	(-0.21, 0.21)	0.985	-0.15	(-10.93, 10.62)	0.978	-0.02	(-0.15, 0.11)	0.755
5-day average[d]	-0.05	(-0.09, 0.00)	0.058	-0.04	(-0.26, 0.17)	0.711	6.58	(-7.16, 20.33)	0.348	0.01	(-0.12, 0.15)	0.871
NO_2												
Test day[a]	-0.01	(-0.05, 0.02)	0.395	-0.16	(-0.40, 0.08)	0.192	-0.75	(-17.35, 15.86)	0.930	0.08	(-0.09, 0.25)	0.350
Previous day[b]	0.01	(-0.02, 0.04)	0.560	-0.06	(-0.29, 0.16)	0.581	-10.01	(-25.94, 5.91)	0.218	0.07	(-0.09, 0.23)	0.396
2-day average[c]	0.00	(-0.03, 0.03)	0.945	-0.11	(-0.36, 0.13)	0.376	-6.28	(-23.45, 10.89)	0.473	0.08	(-0.09, 0.25)	0.357
3-day average[d]	0.00	(-0.04, 0.03)	0.966	-0.12	(-0.42, 0.18)	0.428	-7.74	(-27.70, 12.22)	0.447	0.11	(-0.11, 0.32)	0.329
4-day average[d]	0.01	(-0.03, 0.05)	0.683	-0.22	(-0.59, 0.15)	0.237	3.48	(-18.10, 25.07)	0.752	0.18	(-0.06, 0.43)	0.148
5-day average[d]	0.02	(-0.03, 0.07)	0.393	-0.34	(-0.76, 0.09)	0.119	12.11	(-9.88, 34.09)	0.281	0.25	(-0.03, 0.52)	0.085
SPM												
Test day[a]	-0.02	(-0.03, 0.00)	0.019	-0.02	(-0.08, 0.05)	0.612	-0.03	(-7.92, 7.87)	0.995	-0.04	(-0.09, 0.01)	0.118
Previous day[b]	-0.04	(-0.08, -0.01)	0.008	0.05	(-0.08, 0.18)	0.460	-3.66	(-17.09, 9.77)	0.594	-0.08	(-0.17, 0.02)	0.112
2-day average[c]	-0.03	(-0.05, -0.01)	0.008	-0.01	(-0.08, 0.07)	0.868	-0.96	(-10.73, 8.82)	0.848	-0.05	(-0.11, 0.02)	0.149
3-day average[d]	-0.05	(-0.08, -0.01)	0.004	0.01	(-0.11, 0.13)	0.857	0.59	(-13.35, 14.52)	0.934	-0.08	(-0.18, 0.01)	0.091
4-day average[d]	-0.06	(-0.11, -0.01)	0.021	-0.01	(-0.19, 0.16)	0.870	10.13	(-10.19, 30.46)	0.328	-0.12	(-0.30, 0.07)	0.206
5-day average[d]	-0.05	(-0.10, 0.00)	0.064	-0.06	(-0.24, 0.13)	0.547	17.89	(-3.62, 39.40)	0.103	-0.09	(-0.30, 0.13)	0.448

CI = confidence interval, O_3 = ozone, NO_2 = nitrogen dioxide, SPM = suspended particulate matter

[a] Test day: The 24-hour mean concentration of a pollutant collected on the day that respiratory function tests were done.

[b] Previous day: The 24-hour mean concentration of a pollutant collected on the day before the respiratory function tests were done.

[c] 2-day average: The mean concentration of a pollutant collected on the day and the day before the respiratory function tests were done.

[d] 3- to 5-day average: The mean concentration of a pollutant collected on the day and the 2 to 4 days before the respiratory function tests were done.

All models are adjusted for temperature and relative humidity.
