# Supplementary material for: Acute Effects of Summer Air Pollution on Pulmonary Function and Airway Inflammation in Healthy Young Women
Source: J Epidemiol. 2014 Jul 5;24(4):312–20. doi: 10.2188/jea.JE20130155 (PMC4074636; doi:10.2188/jea.JE20130155)
Supplement: Abstract in Japanese. [file je-24-312-s002.pdf]

Acute Effects of Summer Air Pollution on Pulmonary Function and Airway Inflammation in Healthy Young Women
（夏季の大気汚染が健常若年女性の肺機能及び気道炎症に与える急性影響）

余田佳子[1]、大谷成人[1]、櫻井四郎[2]、島　正之[1]

[1] 兵庫医科大学公衆衛生学
[2] 大妻女子大学社会情報学部環境情報学専攻

背景：大気汚染への曝露が喘息患者の症状悪化と関連していることは数多く報告されている。しかし、健常者に対する大気汚染の急性影響はほとんど知られていない。そこで、健常成人を対象にパネル研究を実施し、肺機能と気道炎症に対する大気汚染物質の急性影響を評価した。
方法:東京都内の健常な女子学生 21 名を対象として、呼気凝縮液（EBC）の pH、呼気一酸化窒素濃度（FeNO）及び肺機能の測定を夏季の 2 週間の間に繰り返し実施した。大気汚染物質の濃度は、対象者が居住する近隣の測定局における浮遊粒子状物質（SPM）及びオゾン（O_3）濃度を用いて、各指標との関連を一般化推定方程式により解析した。
結果： EBC pH は、検査前 4 日平均の O_3 濃度 10ppb 増加あたり-0.07 [95%信頼区間：-0.11,-0.03]、検査前 4 日平均の SPM 濃度 10 μg/m^3 増加あたり-0.08 [95%信頼区間：-0.12,-0.03]であり、いずれも有意な低下が観察された。鼻炎の既往があるものは、O_3 及び SPM 濃度の増加により EBC pH の顕著な低下がみられた。1 秒量（FEV_1）も検査前 3 日平均の SPM 濃度と有意に関連しており、10 μg/m^3 増加あたり-0.09 [95%信頼区間 : -0.17, -0.01] L の低下であった。FeNO は、喘息の既往のあるものにおいてのみ O_3 及び SPM 濃度が増加すると有意に増加した。
結論：研究期間中に大気中の O_3 及び SPM 濃度が上昇すると、EBC pH が有意に酸性になることが明らかとなった。また、SPM が高濃度では FEV_1 の低下との関連があった。鼻炎または喘息の既往があるものは大気汚染物質の影響を受けやすいと考えられた。

キーワード：気道炎症、呼気凝縮液、オゾン、粒子状物質、肺機能
